# 3-(Prop-2-en-1-yl)-2-sulfanyl­idene-1,2,3,4-tetra­hydro­quinazolin-4-one

**DOI:** 10.1107/S1600536812021800

**Published:** 2012-05-19

**Authors:** Rashad Al-Salahi, Mohamed Al-Omar, Mohamed Marzouk, Seik Weng Ng

**Affiliations:** a Department of Pharmaceutical Chemistry, College of Pharmacy, King Saud University, Riyadh 11451, Saudi Arabia; b Department of Chemistry, University of Malaya, 50603 Kuala Lumpur, Malaysia; c Chemistry Department, Faculty of Science, King Abdulaziz University, PO Box 80203 Jeddah, Saudi Arabia

## Abstract

The tetra­hydro­quinazoline fused-ring system of the title compound, C_11_H_10_N_2_OS, is approximately planar (r.m.s. deviation = 0.019 Å). In the crystal, adjacent mol­ecules are linked by N—H⋯O hydrogen bonds, forming a chain running along the *b* axis.

## Related literature

For the synthesis, see: Shiau *et al.* (1990 ▶); Vassilev & Vassilev (2007 ▶).
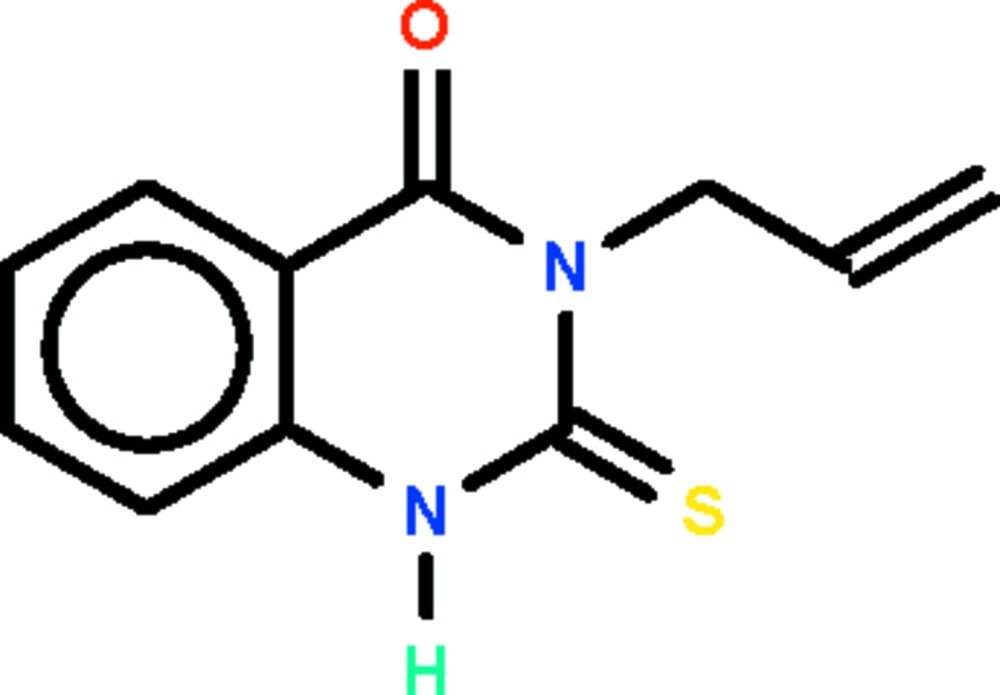


## Experimental

### 

#### Crystal data

C_11_H_10_N_2_OS
*M* *_r_* = 218.27
Monoclinic, 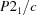

*a* = 8.9823 (3) Å
*b* = 13.7271 (3) Å
*c* = 8.3137 (2) Å
β = 92.882 (3)°
*V* = 1023.79 (5) Å^3^
*Z* = 4
Cu *K*α radiation
μ = 2.59 mm^−1^
*T* = 294 K
0.30 × 0.30 × 0.03 mm

#### Data collection

Agilent SuperNova Dual diffractometer with an Atlas detector
Absorption correction: multi-scan (*CrysAlis PRO*; Agilent, 2012 ▶) *T*_min_ = 0.511, *T*_max_ = 0.927
4913 measured reflections
2128 independent reflections
1855 reflections with *I* > 2σ(*I*)
*R*_int_ = 0.023

#### Refinement

*R*[*F* ^2^ > 2σ(*F* ^2^)] = 0.037
*wR*(*F* ^2^) = 0.108
*S* = 1.06
2128 reflections
140 parameters
1 restraint
H atoms treated by a mixture of independent and constrained refinement
Δρ_max_ = 0.23 e Å^−3^
Δρ_min_ = −0.38 e Å^−3^

### 

Data collection: *CrysAlis PRO* (Agilent, 2012 ▶); cell refinement: *CrysAlis PRO*; data reduction: *CrysAlis PRO*; program(s) used to solve structure: *SHELXS97* (Sheldrick, 2008 ▶); program(s) used to refine structure: *SHELXL97* (Sheldrick, 2008 ▶); molecular graphics: *X-SEED* (Barbour, 2001 ▶); software used to prepare material for publication: *publCIF* (Westrip, 2010 ▶).

## Supplementary Material

Crystal structure: contains datablock(s) global, I. DOI: 10.1107/S1600536812021800/bt5919sup1.cif

Structure factors: contains datablock(s) I. DOI: 10.1107/S1600536812021800/bt5919Isup2.hkl

Supplementary material file. DOI: 10.1107/S1600536812021800/bt5919Isup3.cml

Additional supplementary materials:  crystallographic information; 3D view; checkCIF report

## Figures and Tables

**Table 1:** Hydrogen-bond geometry (Å, °)

*D*—H⋯*A*	*D*—H	H⋯*A*	*D*⋯*A*	*D*—H⋯*A*
N1—H1⋯O1^i^	0.87 (1)	2.15 (1)	2.977 (2)	160 (2)

Symmetry code: (i) 
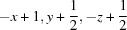
.

**Fig. 1.**
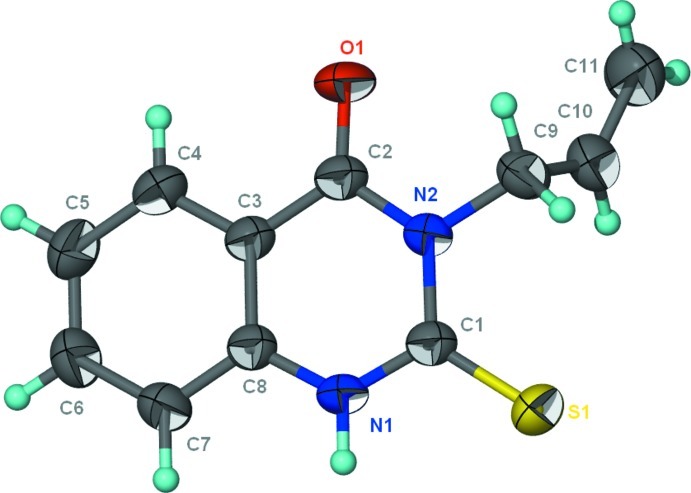
Anisotropic displacement ellipsoid plot (Barbour, 2001) of C11H10N2OS at the 70% probability level; hydrogen atoms are drawn as spheres of arbitrary radius.

**Fig. 2.**
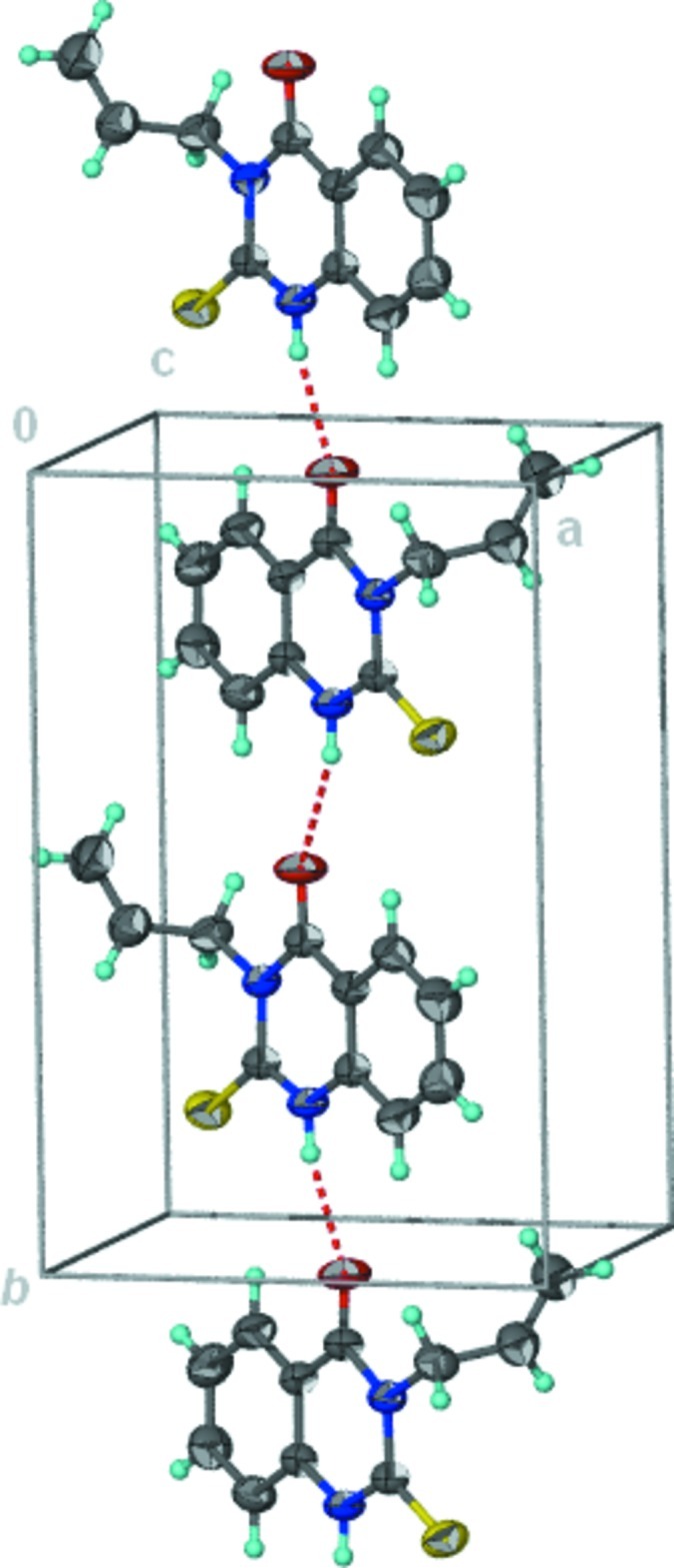
Hydrogen-bond chain structure.

## supplementary crystallographic information

### Comment

The compound, 3-benzyl-8-methoxy-2-sulfanylidene-1,2,3,4-tetrahydroquinazolin-4-one, was previously synthesized for a study of its antimicrobial activity. The related 2-sulfanylidene-1,2,3,4-tetrahydroquinazolin-4-one (Scheme I) exhibits cytokinin activity (Vassilev & Vassilev, 2007). The synthesis described in the present study is a more straightforward procedure than those previously reported (Shiau *et al.*, 1990; Vassilev & Vassilev, 2007). The tetrahydroquinazoline fused-ring of C_11_H_10_N_2_OS is planar (Fig. 1). Adjacent molecules are linked by an N–H···O hydrogen to form a chain running along the *b*-axis of the monoclinic unit cell (Table 1).

### Experimental

Allyl isothiocyanate (10 mmol, 0.99 g), 2-amino-5-methylbenzoic acid (10 mmol, 1.51 g) and triethylamine (5 mmol, 0.51 g) in ethanol (30 ml) was heated for two hours. The mixture was poured into ice-cold water. The solid was collected and recrystallized from ethanol to give colorless crystals.

### Refinement

All H-atom were located in a difference Fourier map. Carbon-bound H-atoms were placed in calculated positions [C–H 0.93 to 0.97 Å, *U*_iso_(H) 1.2*U*_eq_(C)] and were included in the refinement in the riding model approximation.

The amino H-atom was refined isotropically with a distance restraint of N–H 0.88±0.01 Å.

### Crystal data

**Table:**

C_11_H_10_N_2_OS	*F*(000) = 456
*M**_r_* = 218.27	*D*_x_ = 1.416 Mg m^−^^3^
Monoclinic, *P*2_1_/*c*	Cu *K*α radiation, λ = 1.54184 Å
Hall symbol: -P 2ybc	Cell parameters from 2303 reflections
*a* = 8.9823 (3) Å	θ = 3.2–76.4°
*b* = 13.7271 (3) Å	µ = 2.59 mm^−^^1^
*c* = 8.3137 (2) Å	*T* = 294 K
β = 92.882 (3)°	Prism, colorless
*V* = 1023.79 (5) Å^3^	0.30 × 0.30 × 0.03 mm
*Z* = 4	

### Data collection

**Table:**

Agilent SuperNova Dual diffractometer with an Atlas detector	2128 independent reflections
Radiation source: SuperNova (Cu) X-ray Source	1855 reflections with *I* > 2σ(*I*)
Mirror monochromator	*R*_int_ = 0.023
Detector resolution: 10.4041 pixels mm^-1^	θ_max_ = 76.6°, θ_min_ = 5.9°
ω scan	*h* = −11→10
Absorption correction: multi-scan (*CrysAlis PRO*; Agilent, 2012)	*k* = −17→10
*T*_min_ = 0.511, *T*_max_ = 0.927	*l* = −10→8
4913 measured reflections	

### Refinement

**Table:**

Refinement on *F*^2^	Primary atom site location: structure-invariant direct methods
Least-squares matrix: full	Secondary atom site location: difference Fourier map
*R*[*F*^2^ > 2σ(*F*^2^)] = 0.037	Hydrogen site location: inferred from neighbouring sites
*wR*(*F*^2^) = 0.108	H atoms treated by a mixture of independent and constrained refinement
*S* = 1.06	*w* = 1/[σ^2^(*F*_o_^2^) + (0.0596*P*)^2^ + 0.1451*P*] where *P* = (*F*_o_^2^ + 2*F*_c_^2^)/3
2128 reflections	(Δ/σ)_max_ = 0.001
140 parameters	Δρ_max_ = 0.23 e Å^−^^3^
1 restraint	Δρ_min_ = −0.38 e Å^−^^3^

### Fractional atomic coordinates and isotropic or equivalent isotropic displacement parameters (Å^2^)

**Table:**

	*x*	*y*	*z*	*U*_iso_*/*U*_eq_	
S1	0.77201 (5)	0.32274 (3)	0.07911 (5)	0.04374 (16)	
O1	0.54446 (14)	0.00715 (8)	0.22169 (16)	0.0488 (3)	
N1	0.53208 (14)	0.29760 (9)	0.24120 (16)	0.0337 (3)	
H1	0.530 (2)	0.3608 (7)	0.239 (2)	0.052 (6)*	
N2	0.64135 (14)	0.15416 (9)	0.15732 (15)	0.0317 (3)	
C1	0.64308 (17)	0.25505 (10)	0.16333 (17)	0.0317 (3)	
C2	0.53764 (17)	0.09571 (11)	0.23320 (18)	0.0341 (3)	
C3	0.42514 (16)	0.14680 (11)	0.32027 (18)	0.0320 (3)	
C4	0.31809 (19)	0.09589 (12)	0.4035 (2)	0.0404 (4)	
H4	0.3200	0.0282	0.4068	0.048*	
C5	0.2099 (2)	0.14630 (14)	0.4804 (2)	0.0458 (4)	
H5	0.1379	0.1125	0.5345	0.055*	
C6	0.20775 (19)	0.24781 (14)	0.4776 (2)	0.0445 (4)	
H6	0.1339	0.2813	0.5293	0.053*	
C7	0.3140 (2)	0.29896 (11)	0.3990 (2)	0.0393 (4)	
H7	0.3127	0.3667	0.3982	0.047*	
C8	0.42342 (16)	0.24836 (11)	0.32063 (17)	0.0311 (3)	
C9	0.76129 (18)	0.10324 (12)	0.07515 (18)	0.0376 (4)	
H9A	0.7907	0.1420	−0.0155	0.045*	
H9B	0.7242	0.0412	0.0340	0.045*	
C10	0.89456 (19)	0.08610 (13)	0.1881 (2)	0.0433 (4)	
H10	0.9300	0.1382	0.2505	0.052*	
C11	0.9639 (2)	0.00289 (15)	0.2043 (3)	0.0568 (5)	
H11A	0.9313	−0.0506	0.1436	0.068*	
H11B	1.0459	−0.0029	0.2765	0.068*	

### Atomic displacement parameters (Å^2^)

**Table:**

	*U*^11^	*U*^22^	*U*^33^	*U*^12^	*U*^13^	*U*^23^
S1	0.0415 (3)	0.0325 (2)	0.0586 (3)	−0.00599 (15)	0.01623 (19)	0.00349 (16)
O1	0.0559 (7)	0.0207 (5)	0.0712 (8)	−0.0015 (5)	0.0180 (6)	−0.0008 (5)
N1	0.0363 (7)	0.0203 (6)	0.0448 (7)	0.0004 (5)	0.0068 (6)	0.0020 (5)
N2	0.0324 (6)	0.0236 (6)	0.0393 (6)	0.0015 (5)	0.0055 (5)	−0.0004 (5)
C1	0.0328 (7)	0.0252 (6)	0.0369 (7)	−0.0002 (5)	0.0014 (6)	0.0009 (5)
C2	0.0358 (8)	0.0252 (7)	0.0414 (8)	−0.0004 (6)	0.0014 (6)	0.0025 (6)
C3	0.0319 (7)	0.0254 (7)	0.0387 (7)	−0.0019 (6)	0.0015 (6)	0.0016 (5)
C4	0.0406 (9)	0.0305 (7)	0.0505 (9)	−0.0053 (6)	0.0072 (7)	0.0044 (6)
C5	0.0414 (9)	0.0461 (9)	0.0511 (9)	−0.0086 (7)	0.0141 (7)	0.0039 (7)
C6	0.0383 (9)	0.0460 (9)	0.0502 (9)	0.0042 (7)	0.0124 (7)	−0.0006 (7)
C7	0.0407 (9)	0.0301 (7)	0.0477 (9)	0.0047 (6)	0.0086 (7)	0.0007 (6)
C8	0.0308 (7)	0.0265 (7)	0.0358 (7)	0.0001 (5)	0.0009 (6)	0.0020 (5)
C9	0.0408 (9)	0.0316 (8)	0.0413 (8)	0.0052 (6)	0.0103 (7)	−0.0015 (6)
C10	0.0382 (8)	0.0422 (9)	0.0502 (9)	0.0042 (7)	0.0092 (7)	−0.0025 (7)
C11	0.0431 (10)	0.0497 (10)	0.0774 (13)	0.0049 (8)	0.0004 (9)	0.0047 (9)

### Geometric parameters (Å, º)

**Table:**

S1—C1	1.6663 (15)	C5—C6	1.394 (3)
O1—C2	1.2212 (18)	C5—H5	0.9300
N1—C1	1.3487 (19)	C6—C7	1.376 (2)
N1—C8	1.3822 (19)	C6—H6	0.9300
N1—H1	0.869 (9)	C7—C8	1.392 (2)
N2—C1	1.3858 (17)	C7—H7	0.9300
N2—C2	1.4031 (19)	C9—C10	1.502 (2)
N2—C9	1.4799 (18)	C9—H9A	0.9700
C2—C3	1.453 (2)	C9—H9B	0.9700
C3—C8	1.394 (2)	C10—C11	1.305 (3)
C3—C4	1.399 (2)	C10—H10	0.9300
C4—C5	1.377 (2)	C11—H11A	0.9300
C4—H4	0.9300	C11—H11B	0.9300
			
C1—N1—C8	125.05 (13)	C7—C6—C5	120.60 (15)
C1—N1—H1	116.1 (14)	C7—C6—H6	119.7
C8—N1—H1	118.8 (14)	C5—C6—H6	119.7
C1—N2—C2	124.18 (12)	C6—C7—C8	119.37 (15)
C1—N2—C9	118.79 (12)	C6—C7—H7	120.3
C2—N2—C9	116.91 (12)	C8—C7—H7	120.3
N1—C1—N2	116.28 (13)	N1—C8—C7	120.78 (14)
N1—C1—S1	120.42 (11)	N1—C8—C3	118.67 (13)
N2—C1—S1	123.29 (11)	C7—C8—C3	120.55 (14)
O1—C2—N2	119.80 (14)	N2—C9—C10	111.18 (12)
O1—C2—C3	123.95 (14)	N2—C9—H9A	109.4
N2—C2—C3	116.24 (13)	C10—C9—H9A	109.4
C8—C3—C4	119.36 (14)	N2—C9—H9B	109.4
C8—C3—C2	119.46 (13)	C10—C9—H9B	109.4
C4—C3—C2	121.18 (14)	H9A—C9—H9B	108.0
C5—C4—C3	119.84 (15)	C11—C10—C9	124.25 (18)
C5—C4—H4	120.1	C11—C10—H10	117.9
C3—C4—H4	120.1	C9—C10—H10	117.9
C4—C5—C6	120.25 (15)	C10—C11—H11A	120.0
C4—C5—H5	119.9	C10—C11—H11B	120.0
C6—C5—H5	119.9	H11A—C11—H11B	120.0
			
C8—N1—C1—N2	3.4 (2)	C2—C3—C4—C5	−177.85 (15)
C8—N1—C1—S1	−177.21 (12)	C3—C4—C5—C6	−0.9 (3)
C2—N2—C1—N1	−3.4 (2)	C4—C5—C6—C7	−0.4 (3)
C9—N2—C1—N1	−179.29 (13)	C5—C6—C7—C8	0.6 (3)
C2—N2—C1—S1	177.16 (11)	C1—N1—C8—C7	179.38 (14)
C9—N2—C1—S1	1.30 (19)	C1—N1—C8—C3	−0.9 (2)
C1—N2—C2—O1	−179.93 (14)	C6—C7—C8—N1	−179.70 (15)
C9—N2—C2—O1	−4.0 (2)	C6—C7—C8—C3	0.5 (2)
C1—N2—C2—C3	1.0 (2)	C4—C3—C8—N1	178.43 (14)
C9—N2—C2—C3	176.97 (12)	C2—C3—C8—N1	−1.7 (2)
O1—C2—C3—C8	−177.37 (15)	C4—C3—C8—C7	−1.8 (2)
N2—C2—C3—C8	1.6 (2)	C2—C3—C8—C7	178.01 (14)
O1—C2—C3—C4	2.4 (2)	C1—N2—C9—C10	86.69 (17)
N2—C2—C3—C4	−178.57 (14)	C2—N2—C9—C10	−89.46 (16)
C8—C3—C4—C5	2.0 (2)	N2—C9—C10—C11	132.15 (19)

### Hydrogen-bond geometry (Å, º)

**Table:**

*D*—H···*A*	*D*—H	H···*A*	*D*···*A*	*D*—H···*A*
N1—H1···O1^i^	0.87 (1)	2.15 (1)	2.977 (2)	160 (2)

Symmetry code: (i) −*x*+1, *y*+1/2, −*z*+1/2.
